# Anthrax Lethal Factor Cleavage of Nlrp1 Is Required for Activation of the Inflammasome

**DOI:** 10.1371/journal.ppat.1002638

**Published:** 2012-03-29

**Authors:** Jonathan L. Levinsohn, Zachary L. Newman, Kristina A. Hellmich, Rasem Fattah, Matthew A. Getz, Shihui Liu, Inka Sastalla, Stephen H. Leppla, Mahtab Moayeri

**Affiliations:** Laboratory of Parasitic Diseases, National Institutes of Allergy and Infectious Diseases, National Institutes of Health, Bethesda, Maryland, United States of America; The Salk Institute for Biological Studies, United States of America

## Abstract

NOD-like receptor (NLR) proteins (Nlrps) are cytosolic sensors responsible for detection of pathogen and danger-associated molecular patterns through unknown mechanisms. Their activation in response to a wide range of intracellular danger signals leads to formation of the inflammasome, caspase-1 activation, rapid programmed cell death (pyroptosis) and maturation of IL-1β and IL-18. Anthrax lethal toxin (LT) induces the caspase-1-dependent pyroptosis of mouse and rat macrophages isolated from certain inbred rodent strains through activation of the NOD-like receptor (NLR) Nlrp1 inflammasome. Here we show that LT cleaves rat Nlrp1 and this cleavage is required for toxin-induced inflammasome activation, IL-1 β release, and macrophage pyroptosis. These results identify both a previously unrecognized mechanism of activation of an NLR and a new, physiologically relevant protein substrate of LT.

## Author Summary

Anthrax lethal toxin (LT) is a protease which can induce rapid death of macrophages accompanied by activation and release of pro-inflammatory cytokines. The previously identified cellular substrates for this toxin have not been shown to play a role in this rapid cell death. This report identifies a new substrate for LT, and demonstrates that its cleavage by the toxin is required for macrophage death. The substrate, Nlrp1, is a member of a large family of intracellular sensors of danger. These sensors, once activated, form a multiprotein complex called the inflammasome and are essential to the host innate immune response. The mechanism of activation for these sensors is not known. The demonstration of cleavage-mediated activation of Nlrp1 in this study represents the first report on a direct biochemical mechanism for inflammasome activation.

## Introduction

Anthrax lethal toxin (LT) is a key virulence determinant of *Bacillus anthracis*. This bipartite toxin consists of the receptor-binding protein protective antigen (PA) and the metalloprotease lethal factor (LF) [1]. LT injection into experimental animals induces a vascular collapse similar to that occurring during anthrax infections. LT also induces rapid lysis of macrophages from certain inbred rodent strains, but macrophage lysis *in vivo* is not essential for toxin-induced death of mice. LF's only known substrates are the mitogen-activated protein kinase kinases (MEKs 1, 2, 3, 4, 6 and 7). To date, no link between their cleavage and macrophage lysis or animal death has been found [1].

LT induces death in certain inbred and outbred rat strains in as little as 37 minutes [2], [3]. We recently used recombinant inbred rats to map the gene controlling LT sensitivity of both the rats and their macrophages to a region of rat chromosome 10 encoding the NOD-like receptor (NLR) Nlrp1 [3]. The Nlrp proteins are pattern recognition receptors (PRRs) that act as cytoplasmic sensors of danger signals ranging from dividing bacteria to crystalline materials [4]. The activated sensors oligomerize and recruit caspase-1 to a multiprotein complex (the inflammasome). Inflammasome-mediated activation of caspase-1 allows it to cleave the precursors of the inflammatory cytokines IL-1β and IL-18, as well as currently unidentified substrates that cause the rapid lysis of macrophages and dendritic cells (pyroptosis). Unlike other Nlrp sensors that have been shown to be activated by a diverse set of stimuli [4], Nlrp1's only identified activator is LF. The mechanism for activation has not yet been identified for any inflammasome sensor. In this study we report that LF cleaves Nlrp1 and that susceptibility of Nlrp1 to this cleavage dictates sensitivity of macrophages to the pyroptosis induced by this toxin.

## Results

### Anthrax lethal toxin cleaves rat Nlrp1 from LT-sensitive rats near the N-terminus

**Figure 1:**
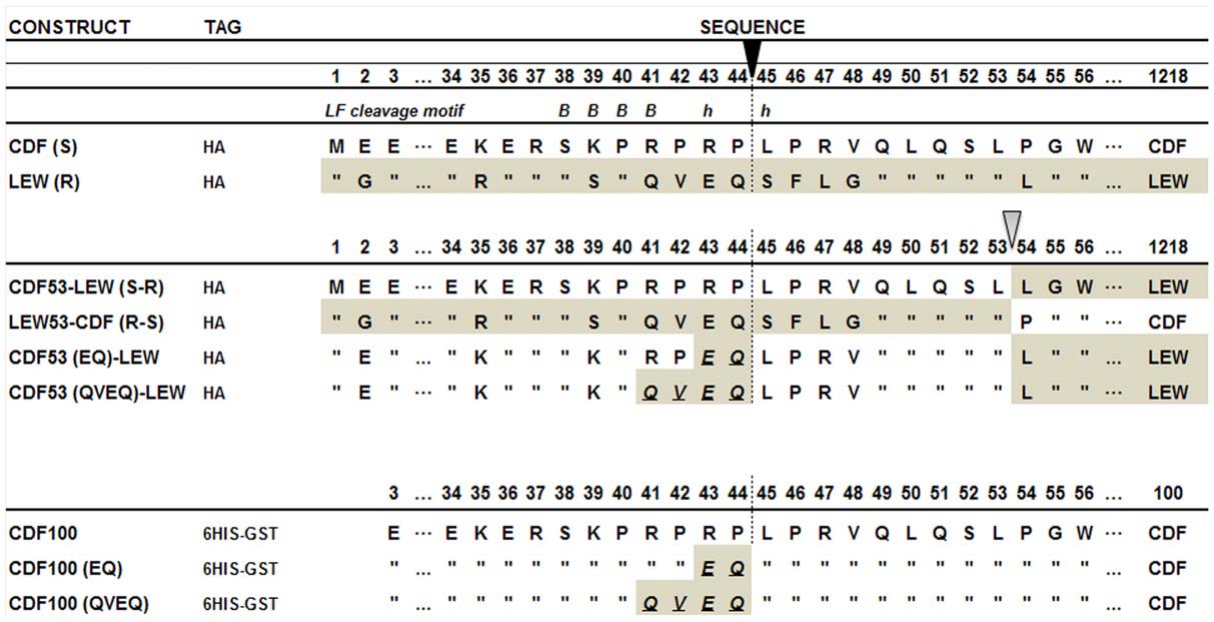
Comparison of the polymorphic regions of the rNlrp1constructs used in this study. The top two constructs represent the full-length HA-tagged rNlrp1 proteins from the LT-sensitive rat (CDF) and the LT-resistant rat (LEW) used in this study. “S” denotes the rNlrp1^S^ sequence and “R” denotes the rNlrp1^R^ sequence. The next four constructs represent chimeric proteins, and chimeric proteins in which mutations were made at the putative LF cleavage site, (shown as a vertical dotted line below the filled arrow). These first six constructs were expressed as full-length proteins in cells. The last three constructs represent proteins where aa 3–100 of rNlrp1were expressed and purified from *E. coli* as 6His-GST tagged proteins. In the sequence alignments, residues identical to those in the construct listed above are indicated by quotations (”). The residue at which fusion of the CDF sequence to LEW or the LEW sequence to CDF occurs is shown with the open arrow. Grey shading denotes LEW sequence, while white denotes CDF sequence. An LF consensus cleavage site motif is shown at the top, where *B* represents basic (positively-charged) residues and *h* stands for hydrophobic residues – the position of each is shown relative to the cleavage site.

**Figure 2:**
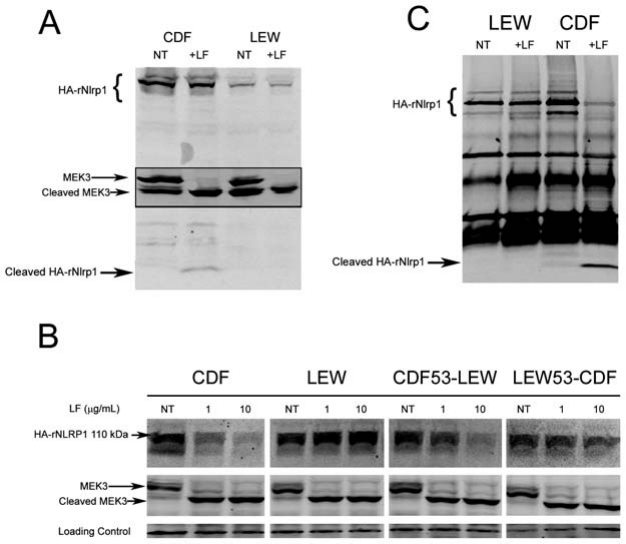
Cleavage of full length rNlrp1 by LF. (A) Treatment of stably-transfected HT1080 cells overexpressing HA-tagged rNlrp1 proteins with LT (1 µg/ml) for 5 h followed by lysis and Western blotting with anti-HA and anti-Mek3 antibodies. Full length and cleaved HA-tagged rNlrp1 are shown. The central panel shows full length and cleaved MEK3 (in a reprobing of the same gel). Cleavage of rNlrp1 leads to appearance of the 6-kDa HA-reactive band. Cleavage of endogenous MEK3 leads to a shift in mobility. (B) Sucrose lysates from stably-transfected HT1080 cells overexpressing HA-tagged rNlrp1 proteins were treated with LF (1 or 10 µg/ml) for 3 h, and subjected to Western blotting with anti-HA (top row) or anti-MEK3 antibody (second row). Cleavage of rNlrp1 leads to loss of the N-terminal HA epitope, and cleavage of endogenous MEK3 leads to a shift in mobility. (C) IP (anti-HA pulldown) followed by anti-HA Western blotting of lysates from HT1080 cells expressing HA-tagged rNlrp1 following treatment with LF (10 µg/ml, 3.5 h). Anti-HA reactive cross-reactive bands not marked as HA-Nlrp1 also appear in vector-transfected controls (data not shown).

Our earlier studies found that polymorphisms within the N-terminus of the rat Nlrp1 (rNlrp1) protein correlate perfectly with LT sensitivity in twelve inbred rat strains [3]. The top two sequences in Fig. 1 are rNlrp1 sequences which represent the previously identified alleles associated with resistance or sensitivity. The CDF Fischer rat (CDF) sequence represents Nlrp1 from LT-sensitive rats (Nlrp1^S^, abbreviated as S in Fig. 1). The Lewis rat (LEW) sequence represents Nlrp1 from LT-resistant rats (Nlrp1^R^, abbreviated as R in Fig. 1). Residues 41–48 of the Nlrp1 from LT-sensitive rats include 3 Arg and 2 Pro residues and are entirely different from the largely hydrophobic residues in the corresponding positions of the Nlrp1 of LT-resistant rats. The other 8 aa differences between the resistant and sensitive Nlrp1 proteins from 12 inbred rat strains are distributed throughout the N-terminal 100 aa [3]. Notably, residues 41–48 of the Nlrp1 from LT-sensitive rats fit consensus cleavage motifs of its MEK substrates [5]–[7] (Fig. 1, top row shows one previously identified consensus motif where the locations of basic (positively-charged) residues (*B*) and hydrophobic residues (*h*) relative to the cleavage site are shown). We hypothesized that LF cleaves Nlrp1 proteins at this site. Nlrp1 proteins are expressed at low levels and are difficult to detect. Therefore we expressed full-length (1218 aa) N-terminally hemagglutinin (HA) epitope-tagged rNlrp1^S^ (CDF), rNlrp1^R^ (LEW), as well as chimeric protein constructs consisting of aa 1–53 of rNlrp1^S^ and aa 54–1218 of rNlrp1^R^ (CDF53-LEW) and the reciprocal construct (LEW53-CDF) (Fig. 1, lines 3,4) in HT1080 human fibroblasts. The proteins were expressed both transiently and in stable lines selected for maximal expression. Western blots and immunoprecipitation (IP) with anti-HA antibodies showed expression of a full length HA-tagged rNlrp1 (140 kDa) in addition to a shorter protein of about 110 kDa (Fig. 2a, 2b, 2c). This smaller protein, which is often present in equal (data not shown) or larger quantities (Fig. 2a, 2b, 2c) than the full-length rNlrp1, probably results from autoproteolysis within the ZU5-UPA/FIND domain that lies upstream of the CARD (caspase-1 recruitment) domain [8]. LT treatment of fibroblasts expressing the HA-tagged rNlrp1 proteins showed a small quantity of a 6-kDa HA-antibody reactive fragment present in LT-treated CDF (Fig. 2a) and CDF-LEW53 cells (data not shown), but not in LEW lines (Fig. 2a), suggesting a possible cleavage event in proteins with rNlrp1^S^ sequences at their N-termini. HT1080 cells expressing any of the three Nlrp1 variants were all equally viable after the LF treatment (data not shown). Cleavage of MEK3 confirmed that LF was active within the cells under the conditions used (Fig. 2a). Direct LF treatment of sucrose lysates of these cells showed loss of the HA-tag from the full length and 110-kDa CDF and CDF53-LEW proteins, but not from the LEW and LEW53-CDF proteins having N-terminal rNlrp1^R^ sequences (Fig. 2b). Loss of HA western blot reactivity from the 110-kDa proteins was consistent with cleavage occurring within aa 1–53, and pointed to the 8-aa polymorphic sequence as being the cleavage site. Anti-HA IP of the toxin-treated lysates showed a single HA-tagged 6-kDa fragment appearing coincidentally with loss of HA-epitope reactivity from both the 140-kDa and 110-kDa rNlrp1 proteins, further supporting the 8-aa sequence as the site of LF action (Fig. 2c). HA-tagged rNlrp1^R^ (LEW) was not cleaved in response to LT (Fig. 2c). An inactive LF variant (LF E687C) was unable to cleave any of the proteins (data not shown).

**Figure 3:**
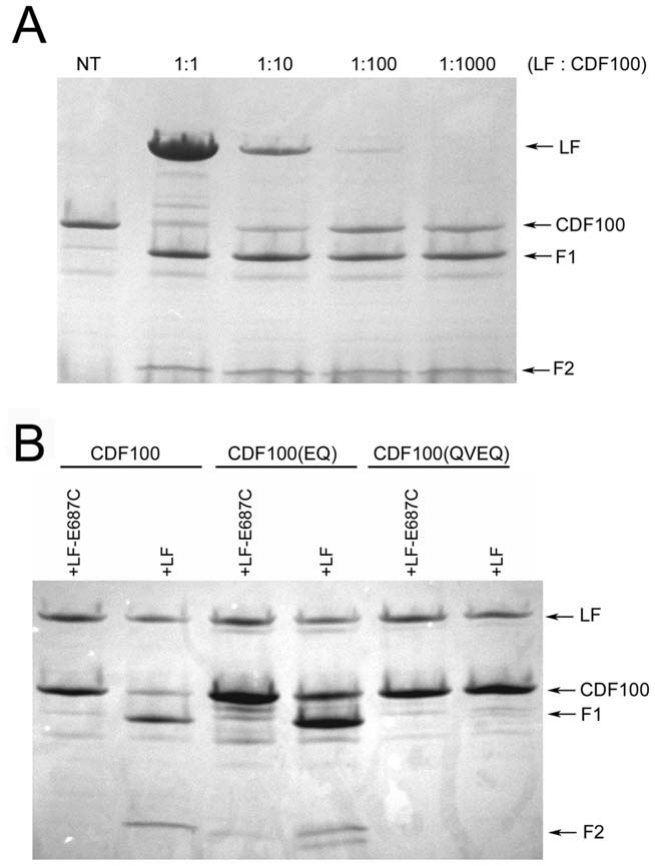
Cleavage of CDF100 by LF. (A) *In vitro* cleavage of N-terminally 6His-GST-tagged aa 3–100 of rNlrp1^S^ (CDF100). Purified protein (1 mg/ml) was treated with the indicated molar ratios of LF for 6 h prior to SDS gel electrophoresis and Coomasie staining. (B) 6His-GST-tagged CDF100 protein or variants (1 mg/ml) were treated with recombinant LF or enzymatically inactive LF E687C (each at 128 µg/ml) for 3 h prior to SDS gel electrophoresis and Coomasie staining. F1 and F2 refer to two fragments generated following LF treatment of CDF100 and its mutated variants.

To precisely identify the cleavage site, we expressed and purified aa 3–100 of the rNlrp1^S^ (designated CDF100) as a fusion containing an N-terminal 6His-GST tag (Fig. 1, line 7). This CDF100 protein was cleaved efficiently even when using an enzyme (LF): substrate (Nlrp1) molar ratio of 1∶1000 (Fig. 3a). Mass spectrometry analyses of the intact protein and the two cleavage fragments yielded protein sizes of 39720, 33260, and 6478 Da (Fig. S1), showing that LF cleaved the Pro44-Leu45 bond in the sequence RPRP∧LPRV of rNlrp1^S^. Variants of the tagged CDF100 protein in which the 2 or 4 aa immediately preceding the LF cleavage site were changed to the corresponding rNlrp1^R^ sequence (CDF100(EQ) and CDF100(QVEQ)) (Fig. 1, lines 8,9) were also tested as substrates for LF or the enzymatically inactive LF variant (LF E687C). LF E687C did not cleave any proteins (Fig. 3b). CDF100(EQ) was cleaved by LF at a lower efficiency than was CDF100 and CDF100(QVEQ) was not cleaved (Fig. 3b). Thus, LF cleaves rNlrp1^S^ at a single site and the specificity of LF for rNlrp1 depends at least in part on the aa sequence immediately preceding the bond cleaved.

### Anthrax lethal toxin cleavage of rat Nlrp1 is required for inflammasome activation and pyroptotic macrophage death

**Figure 4:**
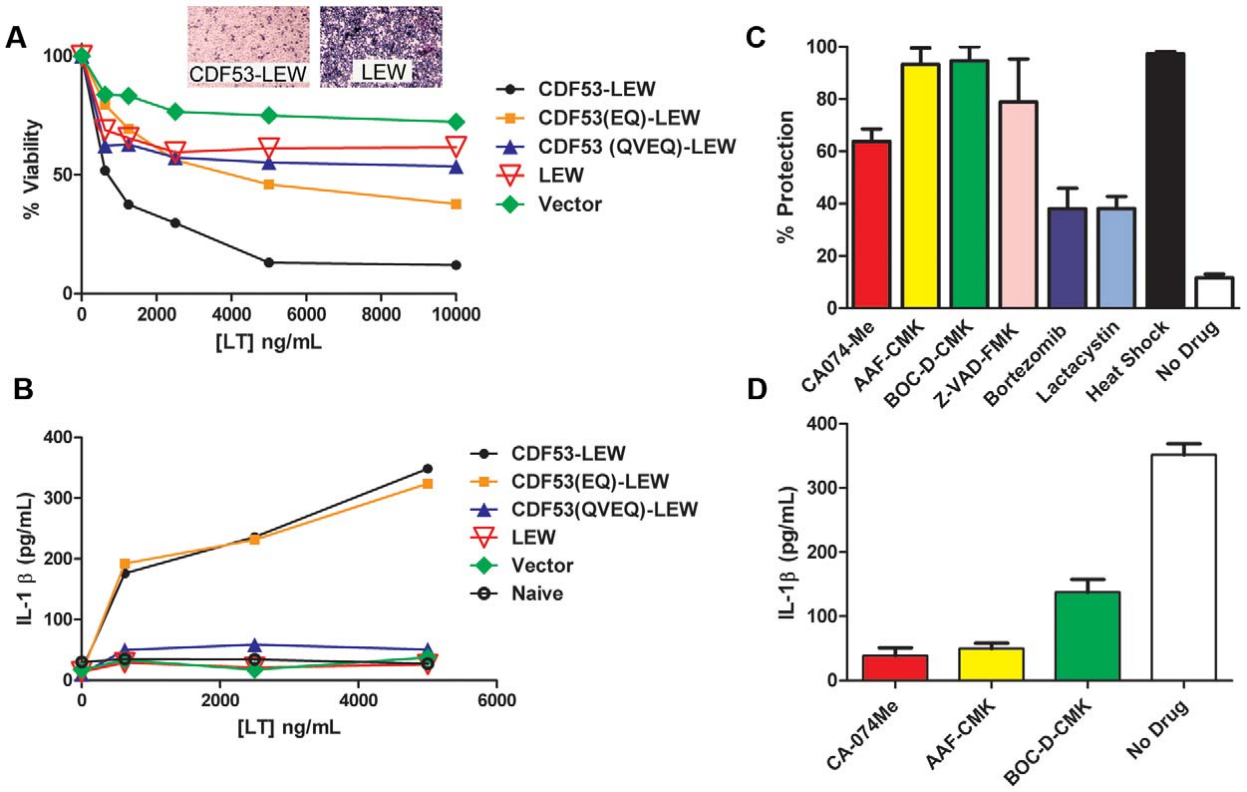
LT effects on cells expressing rNlrp1 constructs. (A) BMAJ cells (normally resistant to LT) that were retrovirally transfected with the indicated rNlrp1 constructs were treated with LT (PA+LF) for 10 h. Percent viability was assessed by MTT staining and calculated relative to untreated controls. Graph shown is from a single experiment, which is representative of eight identically performed experiments. Each point represents the average of three wells. Insets show staining of representative sensitive and resistant cell lines following toxin treatment. (B) Supernatant levels of IL-1β measured by ELISA. Cells were pretreated for 2 h with LPS (0.5–1 µg/ml), prior to LT treatment (7 h). Results shown are from a single experiment, which is representative of three similar experiments. Each point represents the average of duplicate wells in this experiment. (C) Protection of the sensitized BMAJ cell line transfected with rNlrp1fusion CDF53-LEW against challenge with LT (5 µg/ml) following treatment with 50 µM of each drug, or heat shock. Results are average of three experiments, with duplicate wells per treatment in each experiment. (D) Average supernatant IL-1β levels associated with protective treatments from (C) are shown. Results are the average of three experiments, with duplicate wells per treatment in each experiment.

We next asked whether rNlrp1^S^ cleavage is required for LT-mediated macrophage death. Functional inflammasomes containing various NLR proteins have previously been reconstituted in non-macrophage cell lines such as HEK293 cells by expressing the NLR proteins in conjunction with caspase-1 and using IL-1β as a reporter for activation in response to stimuli. Expression of the mouse paralog Nlrp1b and caspase-1 in HT1080 fibroblasts was also shown to be sufficient for induction of approximately 30% cell death following LT treatment [9]. However, we found that robust transient or stable expression of rNlrp1^S^ in conjunction with rat pro-caspase-1 in HT1080 (or CHO WTP4) cells did not sensitize these cells to LT-mediated cell death (data not shown). Because macrophages may express additional components needed for induction of Nlrp1 inflammasome-mediated cell death, we retrovirally expressed various rNlrp1 proteins in the LT-resistant BMAJ mouse macrophage cell line. This cell line expresses the mouse Nlrp1b^R^ protein. Expression of CDF53-LEW in the BMAJ macrophages sensitized them to LT-induced cell death (Fig. 4a). Expression of the CDF53-LEW protein containing the 2 aa substitutions preceding the cleavage site (CDF53(EQ)-LEW) resulted in partial sensitization, while substitution of 4 aa (CDF53(QVEQ)-LEW abrogated sensitization (Fig. 4a). These data strongly implicate the proteolytic cleavage of rNlrp1 by LF as a key step in inflammasome activation and induction of pyroptosis in macrophages. Coincident with cell death, there were equally robust releases of IL-1β from the cells expressing either CDF53-LEW or CDF53(EQ)-LEW (Fig. 4b), in spite of the weaker sensitization of the latter to LT-induced cell death (Fig. 4a). This indicates that there may be different efficiency and timing requirements for the LT-induced caspase-1 activation events that lead to cell death and to IL-1β processing. Supporting this hypothesis, a recent report showed that a single stimulus can be sensed by a single NLR, but activate distinctly different inflammasomes, which activate caspase-1 through different mechanisms [10].

Both mouse and rat Nlrp1 inflammasome activation and the ensuing macrophage death in response to LT can be prevented by a variety of treatments, including thermal shock [11], or inhibition of caspase-1 [12]–[14], cathepsin B [14], [15], and proteasome activity [12], [14], [16]. These treatments all act downstream of LF endocytosis and delivery to the cytosol, as evidenced by their failure to prevent cleavage of the MEK substrates. We tested a panel of these inhibitors for their abilities to protect BMAJ cells expressing CDF53-LEW against LT-induced cell death and to prevent IL-1β release. Protection was provided by nearly all the inhibitors (Fig. 4c,d), the exceptions being the proteasome inhibitors, which are usually highly protective against LT toxicity toward mouse macrophage cell lines [12], [14], [16]. This poor protection may be linked to the longer times (9–10 h) required to achieve complete death of CDF53-LEW-expressing BMAJ cells compared to CDF rat bone marrow-derived macrophages (which succumb in 3 h). The presence of endogenous mouse Nlrp1b^R^ in BMAJ cells likely contributes to the slower death of the transformed cells.

## Discussion

We present here data indicating that cleavage of rNlrp1 by anthrax LT, in an N-terminal domain having no known function, leads to inflammasome activation and macrophage death. This represents the first report on biochemical modification of an inflammasome sensor by a bacterial protein as a mechanism of inflammasome activation. Recent reports demonstrate inflammasome activation mediated by binding of flagellin to NAIP sensor proteins as a simple receptor-ligand activation mechanism that allows for interaction of NAIP proteins with Nlrc4 and caspase-1 in a large oligomer [17], [18]. It remains to be determined how cleavage activates the Nlrp1 inflammasome. Cleavage may induce conformational changes, further proteolytic/autoproteolytic events, release from inhibitory proteins, or alteration in cytosolic localization. Furthermore, it remains to be seen whether cleavage of Nlrp1 directly activates the Nlrp1 inflammasome, or simply allows for subsequent biochemical or cellular events that allow for this sensor or another NLR to recruit and activate caspase-1.

Rodent Nlrp1 proteins differ from most NLRs in that they do not contain a pyrin domain, a domain that is found at the N-terminus of human Nlrp1. This pyrin domain is required for association of NLR proteins with the scaffold protein, ASC, which is required for caspase-1 autoproteolysis and IL-1β processing, but not for caspase-1 mediated cell death [10]. The data presented here raises the question whether mouse or human Nlrp1, or other NLRs, can also be activated by LF through similar cleavage-based mechanisms. Although mouse and human Nlrp1 proteins do not contain the same sequence found in Nlrp1 of sensitive rats, it is possible that LF can also cleave these proteins at a different site in the N-terminal domain or elsewhere. Furthermore, one can speculate that these and other NLR may be activated through cleavage-based mechanisms by other cellular proteases responding to the stimuli associated with inflammasome activation, or even by a conformational change-mediated autoproteolytic event, in a manner similar to caspase-1. An autoproteolytic capability of Nlrp1 was recently reported [8], although a link between this cleavage and inflammasome activation was not demonstrated. It would not be surprising, however, if cleavage of Nlrp1 allowing release of the CARD domain leads to constitutive activation of caspase-1. Expression of the CARD domain of murine Nlrp1 has been shown to result in constitutive caspase-1 activation [9], and deletion of the leucine-rich repeat of Nlrc4 also leads to constitutive caspase-1 activation [17].

This report also identifies a new LF substrate, distinct from the only previously-identified physiologically relevant LF substrates, the MEKs. Although the identification of a substrate with a role in a cell death (i.e., pyroptotic) pathway now explains how the toxin induces rapid macrophage lysis, a role for this pyroptosis in the rapid rat death induced by LT remains to be elucidated.

An increasing number of bacterial virulence factors have been identified which act catalytically to perturb normal cellular processes. In some cases, the catalytic activity mimics activities already present in the cell, as in the case of anthrax edema factor adenylate cyclase, which produces an endogenous signaling molecule, cAMP [19]. In other cases, the bacterial enzyme catalyzes a reaction that inactivates a host process. In the case of the LF-induced cleavage of NLR protein demonstrated here, it is not yet clear whether this cleavage mimics a natural process of inflammasome activation or whether it is an event with no parallel in a normal process. Resolution of this question will come only after there is a better understanding of the mechanisms of inflammasome activation. As has occurred through the study of other bacterial toxins, the further analysis of LF's effects may aid in deciphering the biochemical basis of a key cellular process - in this case, inflammasome activation.

## Materials and Methods

### Ethics statement

This study was carried out in strict accordance with the recommendations in the Guide for the Care and Use of Laboratory Animals of the National Institutes of Health. All bone marrow harvests were from euthanized rats and were performed in accordance to protocols approved by the NIAID Animal Care and Use Committee (approved protocols OSD1, 3).

### Materials

PA, LF, and LF E687C were purified from *B. anthracis* as described previously [20]. Concentrations of LT correspond to the concentration of each toxin component (i.e., 1 µg/ml LT has 1 µg/ml PA and 1 µg/ml LF). 6His-GST-fused rNlrp1 proteins were expressed in plasmid pEX-N-His-GST (cat# PS100028, Origene, Rockville, MD) and purified on glutathione-Sepharose columns by standard methods. High affinity anti-HA (cat# 11867423001, Roche Diagnostics, Indianapolis, IN), anti-Mek3 (cat# sc-959, Santa Cruz BT, Santa Cruz, CA), anti-IL-1β (cat# AF-401-NA, R&D Systems, Minneapolis, MN) and various IR-dye conjugated secondary antibodies (Licor Biosciences, Lincoln, NE and Rockland Immunochemicals, Gilbertsville, PA) were purchased.

### Cell culture and transfections

BMAJ mouse macrophages (gift of D. Radzioch), Phoenix 293T (gift from Iain Fraser, NIAID, NIH), HT1080, and L929 cells were cultured in Dulbecco's Modified Eagle Medium (DMEM) with 10% fetal bovine serum, 10 mM HEPES and 100 µg/ml gentamicin (all obtained from Invitrogen, Carlsbad, CA) at 37°C in 5% CO_2_. CHO WTP4 cells were grown in AMEM with the same supplements. For certain experiments, cells were heat shocked (42°C) for 1 h prior to toxin treatments. For all transfections, endotoxin-free plasmid DNA was prepared (Endo-Free Maxiprep, Qiagen, Valencia, CA) and cells were transfected with TurboFect reagent (Fermentas, Glen Burnie, MD) according to the manufacturer's instructions. For transient transfections, cells were treated with LT 24 h after transfection [3]. Transfected cells were also lysed at the same time point in RIPA buffer and subjected to Western blot (WB) analyses to verify expression of rNlrp1, caspase-1 or IL-1β proteins, as previously described [12]. For stable transfections, HA-rNlrp1 overexpressing cell lines were derived by selection with hygromycin B (500 µg/ml; Invitrogen) for 12 days. High expressing clones were identified by Western blot with anti-HA antibody. For retroviral transduction of macrophages, Phoenix 293T packaging cells were transfected first, as above and at 24 h, culture media were replaced with DMEM. At 48 h, virus-containing media was removed, filtered (0.45 µm membrane, Millipore, Billerica, MA) and supplemented with polybrene at 10 µg/ml (Millipore) prior to incubation with BMAJ macrophages. Virus-containing media were removed 24 h later and replaced with DMEM. Stably-transfected cells were selected over 2–3 weeks in the continuous presence of G418 sulfate (500 µg/ml, Invitrogen). Bone marrow-derived macrophages (BMDMs) used as a source of cDNA were obtained from rats purchased from Charles River Laboratories (Wilmington, MA) and cultured as previously described [12].

### Constructs

cDNA was obtained from different rat strains by isolating RNA from BMDMs [11], [12], [14]. To construct N-terminally hemagglutinin (HA) tagged rNlrp1, an N-terminal HA tag was incorporated into forward primers along with two restriction sites (5′ NheI and 3′ EcoRV- for primer table, see Table S1). For *rNlrp1* from LT-sensitive Fischer (CDF) and LT-resistant Lewis (LEW) rats, full length *rNlrp1* was amplified from CDF cDNA with Ex Taq polymerase (TAKARA Bio Inc., Otsu, Japan) and TOPO TA cloned into the pCR2.1-TOPO vector (Invitrogen). Full length *rNlrp1* was amplified from LEW cDNA with Phusion polymerase (Finnzymes, Woburn, MA) and blunt-end TOPO cloned into the pCR-Blunt II-TOPO vector (Invitrogen). NheI/EcoRV digested fragments were ligated into the pIREShyg3 mammalian expression vector (Clontech, Mountain View, CA). CDF53-LEW- and LEW53-CDF chimeric constructs were made by digestion of CDF-pIREShygV3 and LEW-pIREShygV3 with XcmI/XbaI (New England Biolabs, Ipswich, MA), gel purification of fragments, followed by ligation to yield the appropriate fused constructs. For retroviral expression, 5′ BamHI and 3′ NotI sites were introduced into full length CDF53-LEW and LEW *rNlrp1* amplified from CDF53-LEW-pIREShygV3 and LEW-pIREShygV3 using Phusion polymerase (New England Biolabs). PCR products were cloned into the CloneJET vector (Fermentas) prior to BamHI/NotI digestion and ligation into the pFB-NEO retroviral vector (Agilent Technologies, Santa Clara, CA). QuikChange (Agilent Technologies) was used to introduce 4 and 7 nucleotide changes into CDF53-LEW-pFB-NEO to create CDF53(EQ)-LEW-pFB-NEO and CDF53(QVEQ)-LEW-pFB-NEO. CDF100 was synthesized by Blue Heron Biotechnologies (Bothell, WA) and cloned into the pEX-N-6HIS-GST vector using AsiSI and AscI sites. The sequence of this construct differs from the originally reported CDF rNlrp1sequence at one residue, K61N, resulting in the rNlrp1^S^ allele 1 sequence [3]. The QuikChange system (Agilent Technologies) was used to introduce 4 and 8 nucleotide changes. All clones were identified by restriction digests and verified by sequencing (Macrogen, Rockville, MD).

### Western blots and immunoprecipitation

WB were performed using either anti-HA (1∶1000), anti-MEK3NT (1∶500) or anti-IL-1β (1∶2,500) and proteins were detected using the Odyssey Infrared Imaging System (Licor Biosciences) [11], [12], [14]. For immunoprecipitation (IP) experiments, anti-HA antibody (Roche Diagnostics) was added to each lysate sample (5–15 µg/ml) and samples were continuously mixed by rotation at 4°C for 1 h. Protein A/G agarose (Santa Cruz Biotechnology) was added, and samples were incubated at 4°C overnight with rotation. Beads were centrifuged at 4,000 rpm for 2 min and washed with 10 mM HEPES three times prior to elution of proteins using SDS loading buffer (10% SDS, 0.6 M DTT, 30% glycerol, 0.012% bromophenol blue, at 90°C, 5 min).

### Cleavage assays

To assess Nlrp1 cleavage in cell lysates, cells were grown to confluence in 10-cm tissue culture dishes. For canonical cleavage, cells in two confluent plates were treated with LT, detached by trypsinization, washed 2 times (PBS, 1000 rpm for 5 min) and lysed in 130 µl of sucrose buffer (250 mM sucrose, 10 mM HEPES, 0.05 M EDTA, 0.2% Nonidet-P40) containing 5 ng/ml LF inhibitor PT-168541-1 (gift of Alan Johnson, Panthera Biopharma) followed by addition of 60 µl SDS loading buffer (90°C, 5 min). For cleavage by LF treatment of pre-lysed cells, 5 confluent dishes were detached by trypsinization and washed 3 times (PBS, 1000 rpm for 5 min) and resuspended in 2.5 ml of sucrose buffer (250 mM sucrose, 10 mM HEPES). Cells were centrifuged (2500 rpm, 10 min), resuspended in 350 µl sucrose buffer containing 0.2% Nonidet-P40 and incubated on ice 30 min. All samples were extensively syringed by passage through a 29 gauge hub-less syringe (Terumo, Somerset, NJ) until >99% of all cells were lysed. ZnCl_2_ and NaCl were added to final concentrations of 1 µM and 5 mM, respectively. Lysates were treated with LF for 3.5 h at 37°C. Cleavage was analyzed by WB or IP/WB. For *in vitro* cleavage assays with purified proteins, CDF100, CDF100(EQ) and CDF100(QVEQ) (at final concentrations of 1 mg/ml were incubated in cleavage buffer (1 µM ZnCl_2_, 5 mM NaCl, 10 mM HEPES for 3 h at 37°C) with purified LF at varying concentrations. Samples were separated on an 8–25% SDS-PAGE gel using the PhastSystem (GE Life Sciences, Piscataway, NJ) and stained with Coomassie blue.

### Toxicity and protection assays

All toxicity and protection assays were modified from methods previously described [11], [12], [14] with minor modifications. Drugs were tested over concentrations ranging from 0.1–100 µM and were added 1 h prior to toxin (cathepsin B and caspase-1 inhibitors) or 5 h post-toxin (proteasome inhibitors) treatment. LT treatment was performed with a concentration of 5 µg/ml (9 h). Cell viability was assessed by staining with MTT [3-(4,5-dimethyl-2-thiazolyl)-2,5-diphenyl tetrazolium bromide] as previously described.

### IL-1β measurements

Cells were pretreated with 0.5–1 µg/ml LPS (Calbiochem, San Diego, CA) for 2 h prior to toxin treatment. LT was added for 7 h at varying concentrations. Cytokine in culture supernatants was measured using an IL-1β ELISA kit (R&D Systems) according to the manufacturer's instructions.

### Mass spectrometry

The molecular masses of the H6-GST-LEW and CDF proteins and their cleavage products were determined by liquid chromatography-electrospray mass spectrometry using an HP/Agilent 1100 MSD instrument (Hewlett Packard, Palo Alto, CA) at the NIDDK core facility, Bethesda, MD.

## Supporting Information

Figure S1 Mass spectrometry analyses of cleavage products. Mass spectrometry is shown for the three bands in gel representing uncleaved CDF100 (A), and two cleavage products (B, C).
(DOC)

Table S1 Primers used in this study.
(DOC)
