# Supplementary figures and images for: Anthrax Lethal Factor Cleavage of Nlrp1 Is Required for Activation of the Inflammasome
Source: PLoS Pathog. 2012 Mar 29;8(3):e1002638. doi: 10.1371/journal.ppat.1002638 (PMC3315489; doi:10.1371/journal.ppat.1002638)

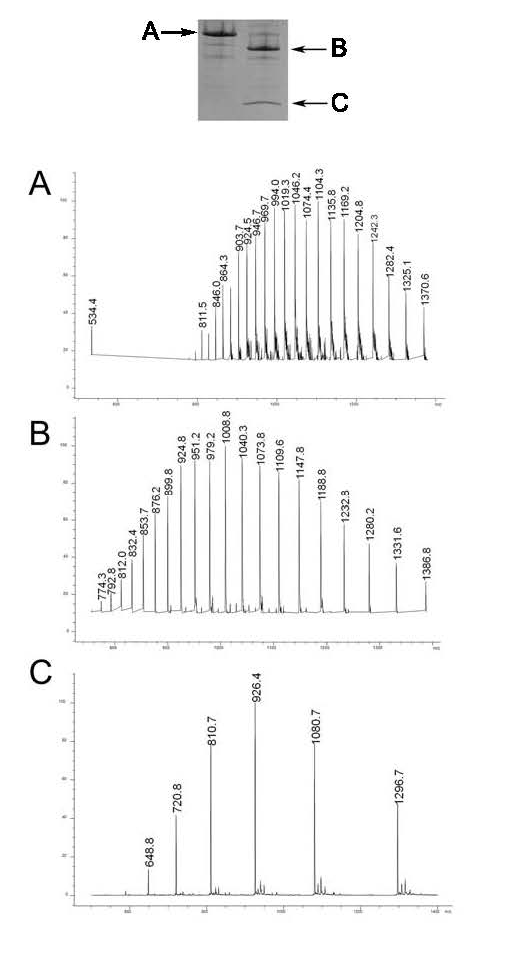

Supplement: Figure S1 — Mass spectrometry analyses of cleavage products. Mass spectrometry is shown for the three bands in gel representing uncleaved CDF100 (A), and two cleavage products (B, C). (DOC) [file ppat.1002638.s001.doc]
