# Supplementary material for: Anthrax Lethal Factor Cleavage of Nlrp1 Is Required for Activation of the Inflammasome
Source: PLoS Pathog. 2012 Mar 29;8(3):e1002638. doi: 10.1371/journal.ppat.1002638 (PMC3315489; doi:10.1371/journal.ppat.1002638)
Supplement: Table S1 — Primers used in this study. (DOC) [file ppat.1002638.s002.doc]

**Table S1.** Primers used in this study.

| **Nlrp1 Sequencing Reaction Primers** | |
| --- | --- |
| **Region 1** | F: GAGAGTCTTGATGCACAAACTTCTCAGAG  R: CAGGCTCTCTTCACCTGCCTGGCCAG |
| **Region 2** | F: CATATGATTGAGATCCAAGACTTAT  R: GCTTCCTCACTATCCTCCAAGATGT |
| **Region 3** | F: TCTGAGCTACAGCTTTGCCCACTTGT  R: ATCCATGTGCTGGAGATGAACAGCT |
| **Region 4** | F: CAGGCCTCTCTCAGTGAGCAGGTGA  R: CACGATATAGCGGGAACCAACATAAAG |
| **Region 5** | F: GGACATCACCTTTCACCTTTACCTG  R: CTGGGTTACACATGGAGAAACTAAG |
| **Cloning Reaction Primers** | |
| **CDF Nlrp1 amplification** | F:AAAAGCTAGCGCCACCATGTACCCATACGATGTTCCAGATTACGCTATGGGAGAATCTCAGTCCAAGCAG  R:AAAAGATATCTCAGAGTCTAACAGAGACCC CTCCCGA |
| **LEW Nlrp1 amplification** | F:AAAAGCTAGCGCCACCATGTACCCATACGA TGTTCCAGATTACGCTATGGAAGAATCTCAGTCCAAGCAG  R:AAAAGATATCTCAGAGTCTAACAGAGACCCCTCCCGA |
| **CDF53-LEW and LEW amplification**  **(for pFB-NEO cloning)** | F:AGGATCCAGCCACCTAGCGCCACCATGTACCCATACGATGTTCCAGATTA  R:TGCGGCCGCTTTTTTATCTCAGAGTCTAACAGAGACCCCTCCCGACTTC |
| **CDF53-LEW(EQ) mutagenesis** | CAAAACCCAGACCCGAGCAGCTTCCTCGGGTGC |
| **CDF53-LEW(QVEQ) mutagenesis** | GAGAAGGAGCGGTCAAAACCCCAAGTCGAGCAGCTTCCTCGGGTGCAGCTCCA |
| **CDF100 (EQ) mutagenesis** | GTTCTAAACCGCGTCCGGAGCAGCTGCCGCGTGTTCAGC |
| **CDF100 (QVEQ) mutagenesis** | AAAAGAACGTTCTAAACCGCAAGTGGAGCAGCTGCCGCGTGTTC |
